# The Impact of China's Paired Assistance Policy on the COVID-19 Crisis—An Empirical Case Study of Hubei Province

**DOI:** 10.3389/fpubh.2022.885852

**Published:** 2022-05-30

**Authors:** Rui Huang, Xiantao Yao, Zhishan Chen, Wan Li, Haobo Yan

**Affiliations:** ^1^Department of Management, School of Management, Minzu University of China, Beijing, China; ^2^Puxin Education and Technology Group, Beijing, China; ^3^Department of Environment and Nature Resources, School of Environment and Nature Resources, Renmin University of China, Beijing, China; ^4^Department of Applied Economics, School of Applied Economics, Renmin University of China, Beijing, China

**Keywords:** COVID-19, paired assistance policy, an empirical case study of Hubei Province, two-way fixed effects model, public health crisis

## Abstract

To control the coronavirus pandemic (COVID-19), China implemented the Paired Assistance Policy (PAP). Local responders in 16 cities in Hubei Province were paired with expert teams from 19 provinces and municipalities. Fully supported by the country's top-down political system, PAP played a significant role in alleviating the COVID-19 pandemic in Hubei Province and China as a whole. In this study, we examined PAP using a two-way fixed effects model with the cumulative number of medical support personnel and cumulative duration as measurements. The results show personnel and material support played an active role in the nation's response to the COVID-19 public health crisis.

## Introduction

As of November 6, 2020, the global cumulative number of confirmed coronavirus disease 2019 (COVID-19) cases exceeded 49.8 million. It continues to increase. But China's total cases have remained below 95,000 for several months. Even in the country's hardest hit city, Wuhan, the pandemic has been effectively controlled. Its crisis response is a model for the world. Throughout the pandemic, the state and all walks of life have invested a tremendous number of resources in Wuhan, with continuously increasing assistance. Other cities in Hubei Province have experienced more severe effects including shortages in medical resources and increasing demands from patients.

The PAP (*Duikou Zhiyuan*) was initially formulated in 1979 as a system and policy in document No. 52. Issued by the Central Committee of the Chinese Communist Party (CCP), it directed the state to “organize the implementation of paired assistance for border areas and ethnic minority areas provided by inland provinces and municipalities” (1). According to the official manual, PAP is an effective form of cross-border cooperation and exchange among regions, industries, and departments. Generally, aid-provider and aid-receiver pairs from these groups are formed to support a certain region or a certain sector while the state formulates macro policies (2). PAP is meant to be “multiple moderated competition” (3). Vertically, Chinese government is a top-down hierarchal system. The central government is the moderator and directs donor local governments to provide financial or personnel support to their affected peers. Horizontally, the donor local governments are competing with each other to provide better crisis response results.

After the COVID-19 outbreak, and in order to ease the emergency situation in Hubei Province, China's National Health Commission (NHC) officially proposed the establishment of one-on-one PAP for Hubei province's cities and prefectures outside of Wuhan. On February 7, 2020, each city in Hubei Province was paired with a province. The goal was to support Hubei Province as a whole, improve the treatment of patients, and safeguard the life and health of the local residents (4). The paired relationships were confirmed in the document “NHC's notice on establishing a paired interprovincial COVID-19 control mechanism of medical aid for regions in Hubei Province outside Wuhan” (5). After the document was issued, various provinces in China responded quickly and organized and dispatched medical aid teams.

But, was PAP the best tool for this case? Did it alleviate the COVID-19 pandemic in Hubei Province? And what aspects of the policy were significantly effective? No research could be found on the effects of PAP on public health outcomes in the COVID-19 pandemic. To fill this gap, we investigated the impact of PAP using econometric models to interpret its effectiveness.

Our work contributes to the field of public management in three ways. First, this study is of critical importance for understanding PAP in the COVID-19 pandemic. Second, our findings show how PAP affected public health results, adding to the recent research on COVID-19 public administration. Finally, an understanding of the effects of PAP on public health outcomes will help government officials make decisions on PAP and administration and help policy makers make more informed decisions.

In this paper, we review the literature on public health emergencies and paired assistance policy. We explore the models that drove our research hypothesis. This includes explaining our research design, describing the variables, and mapping out our empirical strategies. After presenting our empirical results, we also discuss their implications for PAP and administration.

## Literature Review

### Public Health Emergencies

Public health emergencies refer to sudden, unpredictable events that threaten the public's health and safety. These usually manifest as sudden major epidemics of infectious diseases or mass illnesses of unknown origin. Internationally, the study of public health emergencies began in the last century and has led to abundant achievements. The research found that public health emergencies are crises with uncertainty and time pressure that require urgent decisions (6). Western countries have a relatively mature emergency response system for public health emergencies (7). The United States has established a monitoring and early warning system and auxiliary and safeguarding modules for decision-making and the execution of emergency management. The divisions of labor are clear for each department, and they combine to form a three-dimensional and multilayer vertical management system. In the United Kingdom, crisis management has been raised to a strategic level. It is organized by the government to establish a cross-departmental, coordinating mechanism. The management behaviors of those departments are then directed by laws and regulations (8).

After the 2003 severe acute respiratory syndrome (SARS) epidemic, China focused resources on preventing and handling public health emergencies and initiated related research. The government should play a more active role in emergency management and should establish an early warning mechanism (8, 9). From the perspective of social control, various difficulties faced by the government dealing with public emergencies, such as the marketization of resources, the decentralization of rights, and the diversification of systems and stakeholders' interests (10). In the transmission of information on public health emergencies, it is necessary to strengthen the investment in human resources, funding schemes, and supporting facilities in healthcare (11). Simultaneously, emergency management mechanism not only required government leadership but also the extensive participation of various departments and society as a whole (12). However, examined China's relevant documents regarding public health emergencies. It's found that many issues in the practicality and maneuverability of the proposed measures and requirements still remained (13).

After the COVID-19 outbreak at the end of 2019, scholars began to study the influencing factors of COVID-19 spread, the mortality of severe cases, etc. Some scholars have found that factors such as age can significantly affect the spread of the COVID-19 and the rate of severe illness (14). Some scholars have also paid attention to the impact of environmental factors on COVID-19 and found a causal relationship between PM2.5, PM10 and COVID-19 deaths (15). That is, air pollution could promote the spread of COVID-19 and makes the respiratory system more susceptible to this infection (16). Diseases like cardiovascular are also influencing factors. After the patient is infected with COVID-19, the severe disease and fatality rate are higher (14, 17). At the same time, based on the impact of economic activities on the environment, research by scholars has found that economic growth has led to an increase in air pollutants (such as PM2.5 and NO_2_) leading to air pollution, which further affects deaths from COVID-19 (18). In addition, weather conditions, seasons, and non-climatic factors have potential effects on the spread of COVID-19 cases (19). These studies provide a reference for the prevention and control of this public health emergency. However, the key to the prevention and control work is the policy intervention from the government. More research focuses on government policy intervention. COVID-19 in China began in Hubei. Differentiated policies implemented in Hubei and other parts of China providing a scientific reference for the implementation of differentiated policies based on different risks in public health emergencies (20). In addition, China's useful experience in the rapid and efficient control of the COVID-19 also includes the establishment of temporary hospitals, strict isolation (21), and the key role of government dissemination of epidemic information during COVID-19 pandemic (22). Overall, China's response to COVID-19 prevention and control measures confirms that government's strong interventional policies be vital components of public health emergencies control (23).

In short, China's public health incidents has made some progress, which provides the basis for us. But they have all affirmed governmental entities play an important role in dealing with these emergencies, especially during COVID-19 pandemic.

### Paired Assistance

Paired assistance is a governance mechanism with Chinese characteristics for the horizontal transfer of resources and cross-border cooperation. In the 40 years since its inception, the paired assistance policy has played an important role in the development of ethnic minority areas, the Three Gorges Resettlement Project, and various post-disaster reconstruction programs. It was also put forward as a means of developing ethnic minority areas. The early research mainly described the problems related to the aided construction in ethnic areas (24). And believed that in order to invigorate those regional economies, it is necessary to attach importance to economic and technological cooperation and to provide support (25). Because the local development of Tibet and Xinjiang remains a long-standing national goal, studies on paired assistance have also focused on these two areas. Cadres' aid to Tibet has made many achievements but also has certain limitations (26). In the research on paired assistance in Xinjiang, it is found that the paired assistance policy to Xinjiang has promoted the economic development of Xinjiang (27). The comprehensive social development level and the “hematopoietic” ability of the aided areas have been improved (28).

In addition to paired assistance for ethnic minority areas, the paired assistance for horizontal economic aid has also been extensively investigated. Early studies in this field were biased toward “horizontal economic integration” but later focused on “horizontal transfer payment”. Paired assistance brings enormous economic and social benefits capable of promoting the social modernization of minority areas. Different from economic cooperation, paired assistance has “normative” and “mandatory” characteristics (29). For further development, paired assistance needs to consider the economic factors and the benefits for both parties so sustainable development can be ensured (30). Furthermore, an effective supervision and evaluation mechanism should be established, the incentive mechanisms must be sufficient to cause developed regions to want to provide transfer payments and therefore accelerate transfer payment legislation (30, 31).

Studies on PAP in China have been closely tied to the advancement of practices. Starting in the last century, PAP was used for the Three Gorges Reservoir Area and the reconstruction of disaster areas. Many related studies have been published. From the perspective of PAP's historical achievements and the economic condition of the resettlement stage in the Three Gorges Reservoir Area, it's found that tremendous positive effect of PAP on the economic (32) and social development of the reservoir area (33).

PAP for crisis events in China started with post-disaster reconstruction in the areas hit by the 2008 Wenchuan Earthquake. Studies in this field focus on the following aspects. First, PAP models in disaster areas were examined at the macro level to make recommendations for paired assistance schemes from the perspectives of policy, practical difficulties, and finance. By analyzing the challenges experienced by local governments regarding paired assistance and made several recommendations, such as implementing a reconstruction fund supervision system and strengthening control at the macro level (34). Second, this is to discuss the specific implementation of the policy of “one province or municipality helps one hard-hit county” from a case-by-case perspective, and to discuss the specific methods of counterpart assistance from the objective environment of aided construction (35), project construction (36), etc. Third, specific policies for post-disaster paired assistance were studied. It is proposed that assistance can be provided from the perspectives of local support policies, talent support policies, and technology support policies (37).

In terms of public health crisis management, China did not initiate the paired assistance mechanism during the SARS or H7N9 outbreaks. But, after the influenza A virus subtype H1N1 outbreak, it was initiated at a limited scale. After the COVID-19 outbreak, China implemented a large-scale paired assistance policy to Wuhan, which has become a valuable Chinese experience. However, there are few related studies. Studies of paired assistance for major public health emergencies have only been initiated recently and none are comprehensive. Furthermore, due to the accidental and abnormal features of major public health incidents, the available cases and data are very limited. In other words, there has been no empirical research of PAP's effectiveness.

Outside China, scholars continue to assess the effects of PAP. The research found that PAP highlights the important role of collaboration in improving the disaster response capacity. Applying qualitative analyses, Hu et al. analyzed the operation and function of PAPs to improve medical surge capacity in Hubei Province in China during the COVID-19 pandemic. They emphasize the importance of the hybrid modes of coordination at different levels of government and jurisdictions, and the institutionalization of governance mechanisms in order to ensure the efficiency and effectiveness of the multilevel and multiscale crisis response (38).

In summary, various empirical studies of PAP by investigators from different fields indicate it has a positive effect on the aided area, and local governments play an indispensably important role in the policy. However, empirical research of PAP in the field of public health emergency is still lacking. This is due to insufficient data from relevant studies. During the COVID-19 pandemic, China's large-scale paired assistance to 16 cities in Hubei Province represented the first systematic application of the paired assistance policy in a public health emergency. Therefore, an empirical analysis of the effects of this application of PAP will contribute to the research field.

## Theory

The theory of “comprehensive vulnerability management” (39) and “Organizational learning theory” (40) both declare the collaboration and coordination among communities will improve resiliency and reduce negative impacts because they share knowledge and make informed decisions. During an effective community collaboration, the communities share their knowledge and resources in order to make sense of information and interpretations. Comprehensive vulnerability management is linked to recovery and reconstruction, relocation, and redevelopment (31). Comprehensive vulnerability management also reduces the emotional vulnerability that helps people respond and recover from the disaster effectively. The comprehensive vulnerability management paradigm involves major players including political leadership and government agencies, such as housing, environment, commerce, defense, the public health department, etc. Comprehensive vulnerability management emphasizes the significance of governments' collaboration among local governments and central governments who need to work together to reduce vulnerabilities. It also recognizes the non-profit sector as a great contributor of service and assistance.

In addition, the organizational learning theory has enhanced our understanding of information acquisition, information distribution, information interpretation, and organizational memory, which is induced by the uncertainty and disruption caused by disaster and crisis (41). Through community collaboration, attentions are unified and heightened, and new knowledge and information are created, shared, and distributed. As a result, negative outcomes are reduced and mitigated, and community resiliency is improved. Relying on the theory of comprehensive vulnerability management and the organizational learning theory, this paper aims to test whether PAP was effective in reducing the vulnerabilities and negative impacts of COVID-19 in the most affected cities in Hubei province.

This study examines the effect of PAP on disease control in 16 cities in Hubei Province during the COVID-19 pandemic. Given that no confirmed cases were reported in cities other than Wuhan, Hubei Province, before January 21, 2020, and that the last batch of aid workers left the province on April 8, 2020, the time period from January 21 to April 7, 2020, was chosen. The data for this study were collected from the portal website for the Hubei Provincial Government, the local government portal sites for the aided areas, the official websites for charitable administration agencies (or Red Cross offices) in the aided areas, and government portal sites for the aid-providing provinces.

Along with material assistance, aid teams were sent by each province to Hubei. They were mainly composed of three types of personnel: medical workers, management staff, and prevention staff. Their purpose was to ease the shortage of local medical resources. Considering data availability and our research objectives, we chose four indicators—cumulative number of patients, cumulative number of discharged patients, cumulative number of deaths, and fatality rate. We extracted these data from the daily epidemic announcements issued by the portal sites for the Hubei Provincial Government. We used them to measure the actual impact of PAP on the local pandemic situation.

### Policy Variables

On February 10, 2020, the central government's website called for “Marching to Hubei”, which officially launched the NHC's COVID-19 prevention and control mechanism for the interprovincial paired assistance policy. Specific assistance was then provided by 19 provinces (municipalities) to the 16 cities in Hubei other than Wuhan (see Table 1). As shown in Table 1, however, most provinces and municipalities started aid work before the establishment of the mechanism. For example, on January 26, 2020, Shanxi Province dispatched the first batch of medical aid teams to Tianmen and Qianjiang. Only Fujian, Jiangsu, and Guangxi Provinces initiated aid work after the issuance of the paired assistance policy. Teams were dispatched depending on the actual situation of pandemic prevention and control in the aided areas. Therefore, we calculated time period based on the difference between the dates when the policy actually started and ended. In addition to the time of policy implementation, the intensity of policy implementation also had important policy effects.

**Table 1:** Detailed aid situation in 16 cities in Hubei Province.

**Impacted city**	**Donor province**	**Number of days of the PAP**	**Start date of PAP**	**End date of PAP**
Xiaogan	Chongqing, Heilongjiang	60	2020/1/27	2020/3/27
Jingzhou	Guangdong, Hainan	59	2020/1/28	2020/3/27
Yichang	Fujian	56	2020/2/11	2020/4/7
Ezhou	Guizhou	57	2020/1/28	2020/3/25
Enshi	Tianjin	66	2020/2/1	2020/4/7
Huanggang	Shandong, Hunan	54	2020/1/26	2020/3/20
Xiangyang	Liaoning, Ningxia	52	2020/1/29	2020/3/21
Jingmen	Inner Mongolia, Zhejiang	62	2020/1/28	2020/3/30
Xianning	Yunnan	54	2020/1/27	2020/3/21
Shennongjia Forestry District	Hebei	52	2020/1/31	2020/3/23
Suizhou	Jiangxi	44	2020/2/7	2020/3/22
Huangshi	Jiangsu	45	2020/2/11	2020/3/27
Xiantao	Shanxi	62	2020/1/27	2020/3/29
Shiyan	Guangxi	38	2020/2/11	2020/3/20
Tianmen	Shanxi	61	2020/1/26	2020/3/27
Qianjiang	Shanxi	56	2020/1/26	2020/3/22

### Variables

The “cumulative number of aid workers” was used as the independent variable for policy implementation intensity. The donations received by the aided cities from superiors and the public were used to measure the funding situation in the aided areas. Previous studies show population flow has an important impact on the prevention and control of the pandemic. Therefore, we used the date of lockdown implemented by each aided city to measure the population flow of the aided city. After January 23, 2020, when Wuhan was locked down, Ezhou, Huanggang, Xiantao, and Qianjiang were also close. And, over the next 3 days, so were 12 other cities while the paired assistance policy was initiated. Therefore, we used the financial aid provided to and the lockdown duration for each aided city as control variables to ensure a more accurate regression estimation. The data for the related indicators were obtained from the portal websites for the Hubei Provincial Government, the government portal websites, and the official websites for the charitable administration agencies (or Red Cross office) in each aided city.

### Descriptive Statistics

The indicators and data used in this study are described in Table 2.

**Table 2:** Description of the main variables used to evaluate the effect of policy implementation.

**Variable type**	**Variable name**	**Variable symbol**	**Variable meaning**
Dependent variable	Cumulative number of patients discharged from the hospital	*Cumout*	Measures the change in the number of patients discharged from the hospital before and after the implementation of the policy (increase in medical aid teams)
	Cumulative number of deaths	*Cumdeath*	Measures the change in the number of deaths before and after the implementation of the policy (increase in medical aid teams)
	Cumulative number of patients	*Cumcase*	Measures the change in the number of patients before and after the implementation of the policy (increase in medical aid teams)
Policy variable	Fatality rate (%)	*Frate*	Measures the change in the fatality rate before and after the implementation of the policy (increase in medical aid teams)
	Cumulative number of aid workers	*Support*	Measures the intensity of the aid provided to each aided city; the more aid workers dispatched, the higher the aid intensity
	Time (day)	*Time*	Indicates the time period of data collection; 1–78 in chronological order
	Aided city	*City*	Indicates the different aided cities; 1–16
	Implement before the lockdown policy?	*Ifclose*	0/1 dummy variable; 0: implemented before the city lockdown; 1: implemented after the city lockdown
	Amount of donated funds received (yuan)	*Contribution*	Measures the funding situation in each aided city

The descriptive statistics for the variables used in this study are shown in Table 3. The variables are based on the panel data in the time period from January 21, 2020 to April 7, 2020.

**Table 3:** Descriptive statistics for the variables.

**Variable**	**Obs**	**Mean**	**SD**	**Min**	**Max**
Time	1,248	39.5	22.524	1	78
City	1,248	8.5	4.612	1	16
Cumout	1,248	591.179	760.866	0	3,389
Cumdeath	1,248	27.086	32.57	0	129
Frate	1,248	0.026	0.043	0	1
Cumcase	1,248	889.025	874.322	0	3,518
Support	1,248	222.48	297.226	0	1,449
Ifclose	1,248	0.724	0.447	0	1
Contribution	1,248	8.09e+07	9.05e+07	0	3.28e+08

### Analysis of the Effect of the Paired Assistance Policy

After the Hausmann test, we chose to use the fixed effects model. The traditional panel data fixed effects model only considers individual effects. It fails to account for the correlation of residuals in different periods and regions. Its estimation results are biased. And, that bias increases under the influence of time effects. To avoid that, we chose a two-way fixed effects model that accounts for the individual-fixed effects and time-fixed effects to assess the impact of the paired assistance policy on pandemic control in the aided area. The two-way fixed effects model is similar to the effect of the DUAL difference model, and the fixed effects model can partially solve the endogeneity problem caused by omitted variables, especially the endogeneity problem that only changes with time or only changes with region. And with the development of two-way fixed effects model, scholars have modified and improved the model. When the study data was long panel data, it was necessary to control the result of time trend vs. regression results. In order to solve this problem, the time trend variable is introduced into the two-way fixed effects model instead of the time dummy variable. Since then, this method has been tested in many empirical studies. In Elhorst's study of cigarette demand model based on panel data from 46 states of the United States during 1963–1992 in 2014, the study data was long panel data. In this paper, the author adjusted the two-way fixed effects model by using the time trend term instead of the time dummy variables. The results show that the results obtained by using the time trend variable are more accurate than the traditional fixed model (42). At the same time, in the two-way fixed effects model, the purpose of adding time dummy variables is to absorb the impact of unobserved homogeneous shocks in the time dimension, that is, all individuals have a common time factor, such as macroeconomic shocks, fiscal and monetary policies, etc. However, the data used in this paper are daily data, the study is micro. Previous studies have proved that time dummy variables are not applicable to micro data processing, should be replaced by time trend variables (43–45).

Happen each support city in this article as COVID-19 construction before the outbreak of medical basic situation and different administrative districts of the city area is not changing with time of endogenous variables, while climate belongs to the Hubei province over time basic does not change with regional endogenous variables, not through the website to obtain accurate and timely data, but through the fixed effects model. These endogenous problems can be effectively controlled. In addition, the panel data in this paper are long panel data, that is, the time dimension is much higher than the city dimension. And these data with a daily dimension, belongs to microscopic data. Therefore, in the construction of the two-way fixed effects model, we use time trend variable instead of using time dummy variable. The standard format of the two-way fixed effects model is as follows:


$$\begin{array}{l} y_{it}=\beta_{0}+\beta_{1}policy_{it}+{\lambda_{1}D}_{i}{{+\lambda }_{2}T}_{t}+\beta_{n}X_{n}+\varepsilon_{it} \end{array}$$

where *policy*_*it*_ is the policy dummy variable, which is 0 or 1, respectively, for before and after the implementation of the policy in a certain area *D*_*i*_ is the individual dummy variable to fix the individual effects that do not change with time. *T*_*t*_ is the time trend term. *X*_*n*_ is the control variable. The equation after introducing specific indicators is as follows:


$$\begin{array}{l} y_{it}=\beta_{0}+\beta_{1}{\;policy}_{it}+\beta_{2}ifclose+\beta_{3}contribution+\\ \;{\lambda_{1}time}_{t}{+\lambda }_{2}city_{i}+\varepsilon_{it} \end{array}$$

where β_0_ is a constant term β_*i*_ (*i* = 1, 2, 3) is the coefficient of each indicator variable that affects the pandemic situation in the aided area. λ_1_ (*i* = 1, 2, 3.0.0.78) and λ_2_ (*i* = 1, 2, 3.0.0.16) are, respectively, the time-fixed effect and the location-fixed effect. ε_*it*_ is the random error term.

After substituting the data for the corresponding variables, the results are as follows.

As shown in Table 4, the coefficient of determination, *R*^2^, of the regression equation is 0.599, and its joint *F*-test is significant at the 1% significance level. This indicates the reliability of the estimation of the regression equation is high. The cumulative number of patients is significant at the 1% significance level. In other words, the higher the number of aid workers and medical staff screening and detecting suspected patients, the higher the number of patients included in the statistics. The regression results for the amount of donations is significant at the 1% significance level. This means that after receiving donations, local hospitals were able to admit more patients.

**Table 4:** The regression results for the two-way fixed effects model of the influence of aid intensity on the cumulative number of patients.

**Cumcase**	**Coef**.	**SE**	***t***-**Value**	***P***-**Value**	**[95% Conf. Interval]**	**Sig**
Support	0.795	0.043	18.51	0.000	0.711 to 0.879	^***^
Ifclose	37.346	27.236	1.37	0.171	−16.089 to 90.780	
Contribution	0.000	0.000	8.70	0.000	0.000 to 0.000	^***^
Time	10.584	0.588	17.99	0.000	9.429 to 11.738	^***^
Constant	100.379	31.658	3.17	0.002	38.270 to 162.489	^***^
Mean dependent var	889.025	SD dependent var	874.322	
*R*-squared	0.599	Number of obs	1,248.000	
*F*-test	458.069	Prob > *F*	0.000	
Akaike crit. (AIC)	17,856.767	Bayesian crit. (BIC)	17,882.414	

*** P < 0.01, **P < 0.05, *P < 0.1.

As shown in Table 5, the *R*^2^ of the regression equation is 0.569, and its joint *F*-test is significant at the 1% significance level, indicating the cumulative number of aid workers can explain most of the variation in the cumulative number of discharged patients. The regression results show that for each aid worker increase, the cumulative number of discharged patients increased by 0.415 persons, which is significant at the 1% significance level. As the number of aid workers increased, the care and treatment capabilities of local hospitals improved. This meant patients were helped and discharged sooner. The regression results also suggest the impact of the received donations on the number of discharged patients was very significant. Direct cash donations improved local pandemic prevention capabilities in the short term.

**Table 5:** The regression results for the two-way fixed effects model of the influence of aid intensity on the cumulative number of discharged patients.

**Cumout**	**Coef**.	**SE**	***t***-**Value**	***P***-**Value**	**[95% Conf Interval]**	**Sig**
Support	0.415	0.054	7.72	0.000	0.310 to 0.521	^***^
Ifclose	−66.424	34.140	−1.95	0.052	−133.404 to 0.556	^*^
Contribution	0.000	0.000	2.69	0.007	0.000 to 0.000	^***^
Time	17.401	0.738	23.59	0.000	15.954 to 18.848	^***^
Constant	−205.212	39.683	−5.17	0.000	−283.066 to −127.357	^***^
Mean dependent var	591.179	SD dependent var	760.866	
*R*-squared	0.569	Number of obs	1,248.000	
*F*-test	405.770	Prob > *F*	0.000	
Akaike crit. (AIC)	18,420.700	Bayesian crit. (BIC)	18,446.346	

*** *P < 0.01,*

**P < 0.05,

* *P < 0.1.*

As shown in Table 6, the *R*^2^ of the regression equation is 0.565, and its joint *F*-test is significant at the 1% significance level, indicating the reliability of the estimation of the regression equation is high. The coefficient for the number of aid workers is 0.026, which is significant at the 1% significance level. This means that for each aid worker increase, the cumulative number of deaths increased by 0.026 persons, accounting for 0.09% of the total deaths. This is low relative to the overall average. But it also indicates the intensity of the aid had no significant impact on the death toll. The regression results also show the implementation of the lockdown policy did not have a significant impact on the death toll, but the amount of donations played an important role in reducing the cumulative number of deaths.

**Table 6:** The regression estimation results for the two-way fixed effects model of the influence of aid intensity on the cumulative number of deaths.

**Cumdeath**	**Coef**.	**SE**	***t***-**Value**	***P***-**Value**	**[95% Conf. Interval]**	**Sig**
Support	0.026	0.002	13.78	0.000	0.022 to 0.030	^***^
Ifclose	−1.572	1.191	−1.32	0.187	−3.909 to 0.765	
Contribution	0.000	0.000	6.16	0.000	0.000 to 0.000	^***^
Time	0.502	0.026	19.51	0.000	0.452 to 0.553	^***^
Constant	−2.534	1.385	−1.83	0.067	−5.251 to 0.182	^*^
Mean dependent var	27.086	SD dependent var	32.570
*R*-squared	0.565	Number of obs	1,248.000
*F*-test	398.296	Prob > *F*	0.000
Akaike crit. (AIC)	10,045.614	Bayesian crit. (BIC)	10,071.261

*** *P < 0.01,*

**P < 0.05,

* *P < 0.1.*

As shown in Table 7, the *R*^2^ of the regression equation is only 0.019; the fit of this equation is not good. This means the aid intensity and the implementation of the lockdown policy do not affect the fatality rate. Only the amount of donations is significant at the significance level of 10% and this has a certain effect on fatality. In “Relationship between the ABO Blood Group and the COVID-19 Susceptibility” jointly conducted by investigators from Jinyintan Hospital and Wuhan University, they reported the fatality rate may be related to patient blood type. Other scholars have shown the fatality rate is related to age structure. Using data from Hubei, China, as an example, Magazzino et al. validate causal relationships among economic, atmospheric, and COVID-19 indicators. The results confirm that changes in economic activity can significantly affect levels of atmospheric pollutants, which in turn can significantly change the spread of COVID-19 and its fatality rate (46). That is, environmental factors are also important factors affecting the mortality rate of COVID-19. In short, there is no consensus. Combining the regression results in Table 7, we found external medical support cannot significantly change the fatality rate for the patient population. The rate may be affected by internal factors such as patient blood type and social age structure, and the impact of external environmental factors such as air quality.

**Table 7:** The regression results for the two-way fixed effects model of the influence of aid intensity on the fatality rate.

**Frate**	**Coef**.	**SE**	***t***-**Value**	***P***-**Value**	**[95% Conf. Interval]**	**Sig**
Support	0.000	0.000	1.28	0.200	0.000 to 0.000	
Ifclose	−0.003	0.004	−0.95	0.341	−0.010 to 0.004	
Contribution	0.000	0.000	1.91	0.057	0.000 to 0.000	^*^
Time	0.000	0.000	1.38	0.168	0.000 to 0.000	
Constant	0.018	0.004	4.40	0.000	0.010 to 0.026	^***^
Mean dependent var	0.026	SD dependent var	0.043
*R*-squared	0.019	Number of obs	1,248.000
*F*-test	5.991	Prob > *F*	0.000
Akaike crit. (AIC)	−4,465.782	Bayesian crit. (BIC)	−4,445.264

*** *P < 0.01,*

**P < 0.05,

* *P < 0.1.*

In summary, for each additional aid worker sent to the aided area, the results show the cumulative number of patients increases by 0.795 persons, on average; the cumulative number of deaths increases by 0.026 persons, on average; and, the cumulative number of discharged patients increases by 0.415 persons, on average. The reason the increase in the cumulative number of patients is higher than the sum of the cumulative number of deaths and discharged patients is because the increased medical staff improved the admission and treatment capacity of local medical institutions. The greater the aid intensity, the greater its impact on the cumulative numbers of patients and discharged patients in the aided area. But, critically, the intensity of aid has no significant impact on the fatality rate and cumulative number of deaths. When trained aid teams are sent to medical institutions at all levels in the aid areas, the increase in medical testing and examinations reveals more patients suspected of infection and more confirmed cases can be treated. Therefore, the numbers of confirmed cases, admitted patients, and discharged patients go up. The fatality rate and cumulative number of deaths are affected by the local medical level, local demographic characteristics, and other internal factors. Those include the transmission rate of severe acute respiratory syndrome coronavirus 2 (SARS-CoV-2), and the lack of a currently effective treatment for COVID-19. Therefore, the intensity of the aid has no significant impact on the fatality rate and cumulative death toll in an aided area.

Donations received by aided areas impart statistically significant effects on the cumulative numbers of patients and discharged patients and even the fatality rate. According to the data on the distribution of donations received by the local governments, as disclosed through official websites, the cash donations were mostly allocated to local medical institutions to be used for the procurement of personal protective equipment. During the pandemic, medical supplies were in short supply. The donations greatly alleviated the shortage. Given the special nature of public health emergencies, adequate medical supplies play an important role in the fight against a pandemic outbreak.

Jia et al. modeled the mobile data of mobile phone users and analyzed the relationship between the outflow of the population from Wuhan and the pandemic risk level of the areas into which the population flowed (inflow areas) (47). They found population flow had a profound influence on the pandemic situation in the inflow areas. Some scholars predicted the spread of the pandemic using the SEIR [acronym for susceptible (S), exposed (E), infected (I), and resistant (R)] model. It was found the city's lockdown policy significantly reduced population movement and thus significantly controlled the spread of the pandemic. But, in this study, we found that the implementation of the lockdown policy had no significant impact on cumulative numbers of deaths, discharged patients, and patients. The differences are likely because local residents and migrant workers in Hubei Province, the area hardest hit by the pandemic, were more inclined to move out rather than move in. Furthermore, the lockdown policy limited the outflow of some who were infected. The risk of cross-infection among people in the city before the lockdown likely increased. Over the short-term, the number of infected people increased but the curve flattened over the long term. This is consistent with the conclusion of this study.

The paired assistance policy implemented by the Chinese government in response to the pandemic has indeed played an important role in the prevention and control of the pandemic in the aided areas. As more medical aid staff worked in the aided area, medical institutions could admit, treat, and discharge more COVID-19 patients. The fatality rate and cumulative number of deaths in the aided areas did not change significantly. These outcomes were likely affected by internal factors such as the characteristics of SARS-COV-2. For example, temperature can affect the viability of the virus to a certain extent. Air pollution can accelerate virus spread and make the respiratory system more susceptible to infection and complications of coronavirus disease (48), which may have affected the effectiveness of the aid. Donations from the public greatly relieved the shortage of medical supplies, but the impact of the lockdown policy on the effectiveness of the aid was not significant.

### Robustness Test

Given the impact of the population size in each aided area on the number of patients, we further examined the robustness of the model. Using “prevalence” (prevalence = cumulative number of patients in each region/resident population in the region; unit: 1/10,000) to replace “cumulative number of patients,” we tested whether the regression results were still significant. The data were obtained from the *2019 Statistical Yearbook of Hubei Province*.

As shown in Table 8, the *R*^2^ of the regression equation is 0.567, and its joint *F*-test is significant at the 1% significance level, indicating the reliability of the results of the regression equation is high. The prevalence is significant at the 1% significance level, indicating the higher the number of aid workers, the higher the number of medical staff screening patients with suspected cases and the higher the number of patients included in the statistics. The regression results for the amount of donations are significant at the 1% significance level, indicating that, after receiving donations, the local hospitals' ability to admit patients improved. The lockdown policy is also significant at the 5% significance level. The results of the model are highly consistent with those described above, indicating the two-way fixed effects model used in this study can evaluate the effectiveness of the paired assistance policy implemented during the COVID-19 pandemic. And, finally, prevalence removes the effect of population size. This indicates that, regardless of the overall population size and as long as paired assistance in the form of personnel and materials is provided, there will be a positive effect on the diagnosis and treatment of patients per unit population (per 10,000 people). This shows the value of the paired assistance policy from yet another perspective.

**Table 8:** Regression results for the robustness test model of the influence of aid intensity on prevalence.

**Prevalence**	**Coef**.	**SE**	***t***-**Value**	***P***-**Value**	**[95% Conf. Interval]**	**Sig**
Support	0.002	0.000	14.29	0.000	0.002 to 0.003	^***^
Ifclose	0.238	0.100	2.39	0.017	0.043 to 0.434	^**^
Contribution	0.000	0.000	7.57	0.000	0.000 to 0.000	^***^
Time	0.041	0.002	19.13	0.000	0.037 to 0.045	^***^
Constant	0.313	0.116	2.70	0.007	0.086 to 0.540	^***^
Mean dependent var	3.142	SD dependent var	2.775
*R*-squared	0.567	Number of obs	1,248.000
*F*-test	402.396	Prob > *F*	0.000
Akaike crit. (AIC)	3,852.504	Bayesian crit. (BIC)	3,878.150

*** *P < 0.01,*

** *P < 0.05,*

*P < 0.1.

## Policy Recommendations

In emergency management systems for public health emergencies, paired assistance should be used, and a long-term mechanism should be established to do so. In this study, we found the paired assistance policy was significantly effective in responding to the public health emergency. The greater the support intensity, the more profound the effect. Despite this, China has not yet established a complete paired assistance mechanism, and neither have any Western countries. As shown in this study, a paired assistance policy should be included in emergency management systems for public health emergencies. Doing so requires establishing a systematic, long-term mechanism. This means clarifying the starting conditions, scale, time and other aspects, so that paired assistance can play a more effective role in the prevention and control of public health emergencies.

When these incidents arise, we should focus paired assistance on materials and funds. In this study, we found these two forms of tangible support had positive impacts on all aspects of pandemic prevention and control. One of the important differences between public health events and other crises lies in the strong demand for medical supplies. Direct and indirect paired assistance involving medical supplies (provided through financial support) can play a timely and significantly positive role in the public health response.

As for personnel resources, we must give more care and encouragement to responders and establish a complete incentive mechanism. In paired assistance for public health emergencies, the most critical element is medical staff. Proper incentives for these personnel are also necessary but so is psychological counseling, preferential salaries, and welfare and honor policies. Social insurance protections should also be strengthened. In addition, paired assistance experience should be included in employee evaluations and made a bonus item for promotion and professional advancement.

## Data Availability Statement

Publicly available datasets were analyzed in this study. This data can be found at: http://www.hubei.gov.cn/zhuanti/2020/gzxxgzbd/zxtb/202003/t20200312_2179631.shtml; https://www.hbcf.org.cn/nv.html?nid=21fca047-9d40-4103-bb17-5ab0615d66a2; https://tianqi.2345.com/wea_history/57278.htm.

## Author Contributions

RH and XY designed and performed research, analyzed data, and wrote paper. ZC, WL, and HY analyzed data and wrote paper. All authors contributed to the article and approved the submitted version.

## Funding

This work was supported by the National Social Science Foundation of China under Grant Number 16BMZ101.

## Conflict of Interest

The authors declare that the research was conducted in the absence of any commercial or financial relationships that could be construed as a potential conflict of interest.

## Publisher's Note

All claims expressed in this article are solely those of the authors and do not necessarily represent those of their affiliated organizations, or those of the publisher, the editors and the reviewers. Any product that may be evaluated in this article, or claim that may be made by its manufacturer, is not guaranteed or endorsed by the publisher.
